# Lesser Omental Infarction: Clinical Insights and Diagnostic Challenges in a Rare Case of Acute Abdominal Pain

**DOI:** 10.7759/cureus.64099

**Published:** 2024-07-08

**Authors:** Wayne A Martini, Christine O Menias, Jessica Komara

**Affiliations:** 1 Emergency Medicine, Mayo Clinic Arizona, Phoenix, USA; 2 Radiology, Mayo Clinic Arizona, Phoenix, USA

**Keywords:** omental infarction, fat necrosis, epigastric pain, gi radiology, anti-inflammatory agents, necrosis, ischemia, abdominal pain, computed tomography (ct), lesser omentum

## Abstract

Intraperitoneal focal fat infarction (IFFI) is a rare condition characterized by infarction of fatty tissue within the abdominal cavity. Lesser omental infarction, a relatively rare type of IFFI, occurs when there is an infarction of fat within the lesser omentum. Patients typically present with acute abdominal pain that can mimic more serious conditions. This case report highlights the clinical presentation, diagnostic challenges, and management strategies for patients presenting to the emergency department with lesser omental infarction.

A 63-year-old female presented to the emergency department with a chief complaint of epigastric abdominal pain that had been persisting for approximately a week and a half. The pain, which initially seemed like a sore muscle, became increasingly sharp and intermittent, with tenderness upon palpation of the epigastric area. Computed tomography (CT) imaging revealed an omental infarct in the lesser sac with focal inflammation in the fat of the lesser omentum. Through conservative management with analgesics and anti-inflammatory medication, the patient experienced resolution of her symptoms within a few days and had a follow-up with the gastrointestinal team several weeks later.

Lesser omental infarction typically results from compromised blood flow due to torsion or thrombosis, leading to ischemia and necrosis of the fatty tissue. CT imaging is crucial for its diagnosis and reveals fat-density lesions with surrounding inflammatory changes. Conservative management is typically effective, though in rare cases, surgical intervention may be necessary when significant vital signs and electrolyte derangements occur.

## Introduction

Intraperitoneal focal fat infarction (IFFI) encompasses the entire spectrum of conditions characterized by the infarction of fatty tissue within the abdominal cavity. Lesser omental infarction is a rare type of IFFI, involving infarction of fat within the lesser omentum or lesser sac. This peritoneal fold connects the lesser curvature of the stomach and the proximal duodenum to the liver. The clinical presentation of lesser omental infarction often includes acute abdominal pain, which can mimic more common and serious conditions such as acute pancreatitis, perforated peptic ulcer, and complicated cholecystolithiasis [1,2].

The pathophysiology of lesser omental infarction is related to compromised blood flow, most commonly occurring due to thrombosis or torsion. This leads to ischemia and necrosis of the fatty tissue. Imaging plays a crucial role in the diagnosis, with computed tomography (CT) of the abdomen and pelvis with intravenous (IV) contrast often highlighting fat-density lesions with surrounding inflammatory changes. This helps differentiate this pathology from other acute causes of abdominal pain [3-6].

Management of lesser omental infarction varies from conservative treatment with analgesics and anti-inflammatory medications, such as nonsteroidal anti-inflammatory drugs, to surgical intervention in cases of significant vital sign abnormalities or electrolyte derangements. The decision is often guided by the extent of the infarction as well as the patient's response to IV fluids and pain medications [6-8].

This case report aims to highlight the clinical presentation, diagnostic challenges, and management strategies for a patient who presented to our emergency department and was ultimately diagnosed with lesser omental infarction. It emphasizes the importance of accurate imaging and awareness of this rare condition among clinicians.

## Case presentation

A 63-year-old female presented to the emergency department for evaluation of epigastric abdominal pain that had been persistent for the last week and a half. She described the pain as initially feeling like a sore muscle, but in the last day, it had become sharper and intermittently she felt waves of pain. She noted that the pain had localized to the epigastric area, did not radiate, and she was able to elicit tenderness with palpation and movement. She denied any nausea, vomiting, change in stool, hematochezia, dysuria, fevers, or chills. She had been tolerating oral intake without any nausea but reported feeling an increased amount of pain when she ate. She noted in the last day or two she had early satiety. She denied any history of similar symptoms, recent travel, illness, or new medication changes. She was taking ibuprofen daily; however, she limited herself to only two tablets of 200 mg each per day.

**Figure 1:**
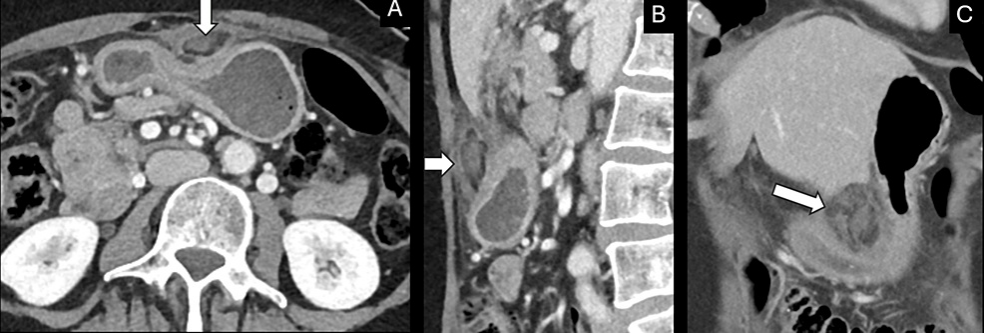
Lesser omental infarction. Axial (A), coronal (B) and sagittal (C) post-contrast CT images demonstrate a focal region of inflammation centered in the fat of the lesser sac/omentum.

On physical examination, the abdomen was soft with tenderness in the epigastric region. She showed no signs of peritonitis. She declined pain medication upon arrival at the emergency department and throughout her stay. Her labs were overall felt to be unremarkable for any acute abnormalities. Her glucose was only slightly elevated at 146 mg/dL (reference range 70-140 mg/dL). Her urinalysis showed ketones; however, it did not show any glucosuria, leukocyte esterase, or nitrite. Her hemoglobin was noted to be stable at 12.0 g/dL (reference range 11.6-15.0 g/dL), and there were no signs of leukocytosis with a white count of 6.7 x 10^9^/L (reference range 3.4-9.6 x 10^9^/L). CT of the abdomen and pelvis with IV contrast showed no bowel wall thickening or obstruction but did reveal a mild thickening of the gastric antrum and focal inflammation of the omental fat of the lesser sac with adjacent peritoneum thickening secondary to inflammation (Figure 1). She remained hemodynamically stable and afebrile throughout her course.

One week following her visit to the emergency department, she had an appointment with Gastroenterology and Hepatology. Her pain had resolved within a day or two of the emergency department visit. She was instructed to take a 10-day course of pantoprazole and to discontinue the use of ibuprofen due to concerns about gastritis and/or peptic ulcer disease as a possible concomitant etiology. At the follow-up visit, she did not experience any gastrointestinal complaints, and she noted significant improvement in her early satiety. She was scheduled for a colonoscopy and upper endoscopy for further evaluation and screening.

## Discussion

Strengths and limitations 

This case report of a 63-year-old female who presented to the emergency department and was ultimately diagnosed with lesser omental infarction adds valuable insight into the diagnosis and management of this rare condition. It reviews a detailed clinical presentation and comprehensive imaging findings and underscores the importance of considering lesser omental infarction in the differential diagnosis of acute epigastric abdominal pain. Lastly, it contributes to the growing body of literature that supports nonsurgical treatment for stable patients.

A limitation of this case report is the lack of a clear etiology for the infarction in this patient. Further investigation into possible predisposing factors, such as hypercoagulable disease, intermittent atrial fibrillation, or other underlying conditions, may enhance understanding. Larger studies, additional case reports, and case series would be beneficial in further understanding the variability in presentation and outcomes of this condition.

Medical literature discussion 

Lesser omental infarction is a rare subtype of IFFI, often presenting with acute abdominal pain that can mimic more serious conditions such as acute pancreatitis, perforated peptic ulcer, and complicated cholecystolithiasis. The pathophysiology involves compromised blood flow to the fat within the lesser omentum, potentially due to torsion or thrombosis, leading to ischemia and necrosis. CT imaging is critical for diagnosis, typically revealing fat-density lesions with surrounding inflammatory changes.

Previous studies have demonstrated that conservative management, including the use of analgesics and anti-inflammatory medications, is often sufficient for patients with lesser omental infarction, provided there are no signs of complications or clinical deterioration. This case supports these findings as the patient’s symptoms resolved with conservative treatment without the need for surgical intervention.

The natural history and epidemiology of omental and epiploic appendage infarction are self-limited in nature; often, treatment involves conservative care, and it is possible to avoid unnecessary surgical interventions [1]. The typical finding of intraperitoneal fat infarction, including lesser omental infarction, is the presence of well-defined fat-density lesions within the lesser omentum. These lesions are surrounded by inflammatory changes, such as fat stranding and increased attenuation, and can involve reactive fluid collections as the body responds to necrosis [2,3]. Occasionally, these lesions may be encapsulated with surrounding reactive fluid, helping to differentiate them from other acute abdominal conditions such as abscesses or tumors [3].

Case series note similarities in patient presentation, with the most frequent symptoms being sharp, constant, and localized pain without significant radiation. Physical examination findings often include localized tenderness without peritoneal signs, which helps differentiate it from conditions that cause generalized peritonitis. Imaging, particularly CT, plays a crucial role in diagnosis, showing inflammatory changes as described above. Conservative treatment with analgesics and anti-inflammatory medications has been found effective in many cases; however, several patients required surgical exploration when the diagnosis was uncertain or when there were signs of complications such as abscess formation or bowel obstruction [8].

Despite the growing body of evidence, challenges remain in the timely and accurate diagnosis of lesser omental infarction. Clinicians must maintain a high index of suspicion and utilize appropriate imaging techniques to differentiate lesser omental infarction from other acute abdominal conditions. Further research is needed to better understand the pathophysiology, optimal management strategies, and long-term outcomes of patients with lesser omental infarction.

## Conclusions

This case report highlights the importance of considering lesser omental infarction in patients who present to the emergency department with a chief complaint of acute epigastric abdominal pain, especially when imaging reveals characteristic fat-density lesions with surrounding inflammatory changes. Accurate and timely diagnosis using CT imaging can prevent unnecessary surgical interventions and guide appropriate conservative management. Clinicians should maintain a high index of suspicion for lesser omental infarction to avoid missed diagnoses and ensure optimal patient outcomes.
